# Direct measurement and modeling of intraglottal, subglottal, and vocal fold collision pressures during phonation in an individual with a hemilaryngectomy

**DOI:** 10.3390/app11167256

**Published:** 2021-08-06

**Authors:** Daryush D. Mehta, James B. Kobler, Steven M. Zeitels, Matías Zañartu, Emiro J. Ibarra, Gabriel A. Alzamendi, Rodrigo Manriquez, Byron D. Erath, Sean D. Peterson, Robert H. Petrillo, Robert E. Hillman

**Affiliations:** 1 Center for Laryngeal Surgery and Voice Rehabilitation, Massachusetts General Hospital, Boston, MA, USA; 2 Department of Surgery, Massachusetts General Hospital–Harvard Medical School, Boston, MA; 3 Speech and Hearing Bioscience and Technology, Division of Medical Sciences, Harvard Medical School, Boston, MA, USA; 4 MGH Institute of Health Professions, Boston, MA, USA; 5 Department of Electronic Engineering, Universidad Técnica Federico Santa María, Valparaíso, Chile; 6 Institute for Research and Development on Bioengineering and Bioinformatics, National University of Entre Rios–CONICET, Entre Ríos, Argentina; 7 Department of Mechanical & Aeronautical Engineering, Clarkson University, Potsdam, NY, USA; 8 Department of Mechanical and Mechatronics Engineering, University of Waterloo, Ontario, Canada

**Keywords:** subglottal pressure, intraglottal pressure, vocal fold collision, hemilaryngectomy

## Abstract

The purpose of this paper is to report on the first *in vivo* application of a recently developed transoral, dual-sensor pressure probe that directly measures intraglottal, subglottal, and vocal fold collision pressures during phonation. Synchronous measurement of intraglottal and subglottal pressures was accomplished using two miniature pressure sensors mounted on the end of the probe and inserted transorally in a 78-year-old male who had previously undergone surgical removal of his right vocal fold for treatment of laryngeal cancer. The endoscopist used one hand to position the custom probe against the surgically medialized scar band that replaced the right vocal fold and used the other hand to position a transoral endoscope to record laryngeal high-speed videoendoscopy of the vibrating left vocal fold contacting the pressure probe. Visualization of the larynx during sustained phonation allowed the endoscopist to place the dual-sensor pressure probe such that the proximal sensor was positioned intraglottally and the distal sensor subglottally. The proximal pressure sensor was verified to be in the strike zone of vocal fold collision during phonation when the intraglottal pressure signal exhibited three characteristics: an impulsive peak at the start of the closed phase, rounded peak during the open phase, and minimum value around zero immediately preceding the impulsive peak of the subsequent phonatory cycle. Numerical voice production modeling was applied to validate model-based predictions of vocal fold collision pressure using kinematic vocal fold measures. The results successfully demonstrated feasibility of *in vivo* measurement of vocal fold collision pressure in an individual with a hemilaryngectomy, motivating ongoing data collection that is designed to aid in the development of vocal dose measures that incorporate vocal fold impact collision and stresses.

## Featured Application

The overall goal of this work is to better understand how vocal fold collision contributes to the development and clinical management of vocal pathologies, such as vocal fold nodules and polyps, and to ultimately develop measures that will improve the prevention, diagnosis, and treatment of phonotraumatic voice disorders.

## 1. Introduction

Voice disorders have been estimated to affect approximately 30% of the adult population in the United States at some point in their lives, with up to 7.6% of individuals affected at any given point in time [1, 2]. The most common voice disorders are chronic or recurring conditions believed to result from excessive and/or poorly regulated activity of the perilaryngeal muscles, referred to as vocal hyperfunction. Vocal hyperfunction can be associated with either phonotrauma-induced lesions of the vocal folds (e.g., nodules and polyps) or with dysphonia occurring in the absence of vocal fold trauma, structural, or neurological abnormalities (e.g., primary muscle tension dysphonia) [3]. The etiology of phonotraumatic lesions has classically centered on the role of vocal fold impact stress (in the direction of tissue motion at contact) and shear stress (along the tissue surface) during phonation [4]. Insight into the role of collision pressures in the generation of vocal fold lesions is important for any quantification or modeling of phonotrauma since phonotraumatic lesions are widely considered to form due to repetitive stress on the mid-membranous portion of the vocal folds at the location of maximal tissue contact [5–7].

The direct measurement of vocal fold collision pressure is challenging to carry out *in vivo*, and only two published studies have attempted to gather data from sensors placed intraglottally during phonation [8, 9]. Verdolini *et al*. [9, 10] developed a piezoresistive pressure sensor with a flat frequency response up to 50 kHz and a linear sensing range of 0–14 kPa (0–140 cm H_2_O). The circular pressure-sensing element was 1.8 mm in diameter (0.4 mm thickness) and inserted transorally via a curved cannula. Limited *in vivo* data were successfully obtained from vocally healthy individuals given challenges related to sensor positioning between two vibrating vocal folds without disrupting phonation and uncertainty as to whether the pressure signal was related to vocal fold collision or acoustic (non-impact) pressures. Vocal fold collision pressures across study participants were reported to be in the range of 4–32 cm H_2_O. However, the criteria by which collision pressure signals in that study were deemed to be of adequate quality were unclear. Only one exemplary waveform exhibited peaks in the pressure signal at the expected vocal fold oscillation rate; but noise in the signal (and a flat-line microphone signal) prevented the ability to confirm whether the peaks were due to impact stress sensing or subglottal (or supraglottal) acoustic pressure sensing.

Gunter *et al*. [8] employed a piezoelectric, force-sensitive plate (10 mm × 15 mm for a sensing area of 150 mm^2^; 0.29 mm thickness) at the end of a transorally positioned rigid cannula that was inserted between the vocal folds. The sensing element exhibited a flat frequency response up to 25 kHz and a linear sensing range for measured forces from a 2.5 mN noise floor up to 200 mN (16–1333 Pa, or 0.16–13.6 cm H_2_O). In that study, peaks in the electroglottography signal and force sensor signal provided an indication of whether the force peaks measured were due to air pressure (force sensor peak lagging the electroglottography peak) or vocal fold collision forces (force sensor peak aligned with the electroglottography peak). Intraglottal placement of the force sensor during phonation was considered satisfactory when endoscopic imaging confirmed the desired sensor positioning against one vocal fold, anterior-posterior positioning in the mid-membranous glottis, and inferior-superior positioning of the force sensor in the coronal plane of vocal fold contact (the *strike zone*). The exemplary waveforms in that paper displayed a force sensor output that resembled a triangular waveform that has not been observed in computational or bench-top models of phonation. The authors acknowledged that the force sensor signal could also reflect acoustic or aerodynamic pressure variations due to the size of the sensor extending above and below the plane of vocal fold contact.

Both of these *in vivo* studies demonstrated the critical role of endoscopic image guidance to aid in the verification of intraglottal sensor placement. In practice, these types of experiments require skillful ambidexterity to position two probes at the same time (one hand holding the intraglottal sensor probe and the other holding the imaging endoscope) and to accurately position the intraglottal sensor with minimal tactile feedback from the laryngeal tissue while viewing a two-dimensional video image; laryngologists develop these skills to perform office-based transoral vocal fold injections to treat vocal fold paralysis and other pathologies. Equally challenging is having to take into account that the coronal plane of vocal fold collision is higher (more superior) during phonation than when the vocal folds are at rest during breathing. Relying solely on visual feedback potentially misses capturing the true intraglottal pressure signal during phonation. Multi-channel techniques using data from multiple pressure sensors can aid in the real-time monitoring of intraglottal positioning; e.g., multi-channel electroglottography applies a similar concept to monitor and verify correct electrode positioning at the glottal level in real time [11]. Essentially, if the signals captured by two adjacent pressure sensors are in phase and correlated, then they are both positioned in the same location—either supraglottally, subglottally, or intraglottally. If the two sensor signals have waveshapes distinct from each other, their difference signal, polarity, and degree of correlation helps confirm that one of the sensors is positioned as desired between the glottis and in the strike zone of collision during phonation [12, 13].

Expectations with respect to the waveform shape of the intraglottal pressure signal come from numerical models of phonation [14–20], self-oscillating physical models of synthetic vocal fold–like material [12, 14, 21], aerodynamically driven excised larynges or hemilarynx models [13, 22–28], and *in vivo* animal models [29]. For example, in a hemilarynx model, the superior-inferior position of a pressure sensor was systematically varied as the model was driven into self-sustained oscillation with an external airflow source [13]. The position of the sensor was confirmed to be in the strike zone of vocal fold collision using a dual-imaging technique that provided *en face* imaging of the medial vocal fold surface and top-down imaging of the vibrating vocal fold. Taken together with the other modeling results, this body of literature provides strong evidence that the *in vivo* intraglottal pressure signal during phonation should have three primary components: (1) an impulsive peak in the direction of increasing pressure at the start of the phonatory closed phase (collision/impact component), which is followed in time by (2) a more rounded peak during the phonatory open phase (acoustic/aerodynamic pressure component) and (3) a minimum value around zero immediately preceding the impulsive peak of the subsequent phonatory cycle. To date, these expected characteristics of the intraglottal pressure waveform have not been directly observed in human speakers.

Direct measurement of vocal fold collision pressures during bilateral vocal fold phonation has proven to be challenging *in vivo* [8, 9]. Positioning a sensor against one vocal fold in a typical larynx can result in disrupted vocal fold vibration and irregular glottal closure characteristics. However, these issues can be mitigated in a group of individuals who have undergone surgical treatment for laryngeal cancer but still exhibit functional voice outcomes due to the cancer being largely limited to one vocal fold [30–32]. This treatment involves performing an endoscopic hemilaryngectomy to remove the primarily affected vocal fold, while preserving as much as possible of the healthy tissue and pliability of the superficial lamina propria on the contralateral side (and, thus, preserve the potential for vocal function). The side with the vocal fold removed is then surgically reconstructed to form a scar band that is medialized using implantable materials (e.g., adipose tissue) so that the vibrating contralateral vocal fold can achieve glottal closure during phonation. The reconstructed larynx is analogous to the excised hemilarynx setup that has been previously employed to study phonatory physiology [e.g., 13, 33, 34, 35]. Many of these patients have perceptually typical conversational voices that are much improved compared with their pre-surgical condition, when they often exhibit insufficient glottal closure that produces excessively inefficient voice production and a breathy voice quality [36].

The primary aim of this paper is to report on the first *in vivo* application of a recently developed transoral, dual-sensor intraglottal/subglottal pressure (ISP) probe [12, 13] in such an individual with a hemilaryngectomy. In these individuals, the tip of the ISP probe is designed to rest against the surgically medialized scar band to yield stable and reliable collision pressure measurements of the medial surface of the contralateral vocal fold (essentially acting as an excised hemilarynx configuration). The probe was designed to simultaneously measure the intraglottal pressure signal from a proximal sensor and subglottal pressure from a second, distal sensor at the tip of the probe to gain empirical insight into *in vivo* vocal fold collision and aerodynamic relationships during phonation. When positioned appropriately during phonation, the intraglottal pressure signal is expected to consist of the two expected components of an impulsive peak due to vocal fold collision and a more rounded peak due to intraglottal aerodynamic pressures during the open phase of the phonatory cycle.

A secondary aim of this paper is to illustrate how numerical models of phonation can be validated using the *in vivo* measurement of vocal fold collision pressures as reference values. Numerical models allow clinicians and researchers to derive hard-to-measure parameters related to voice production (such as vocal fold collision pressures) from relatively easier-to-measure parameters (such as glottal area waveforms) since invasive direct measurements are generally not feasible in practice. Two numerical models will be evaluated that enable the derivation of vocal fold collision pressures from high-speed videoendoscopic imaging data.

## 2. Materials and Methods

### 2.1 Participant characteristics

**Figure 1.**
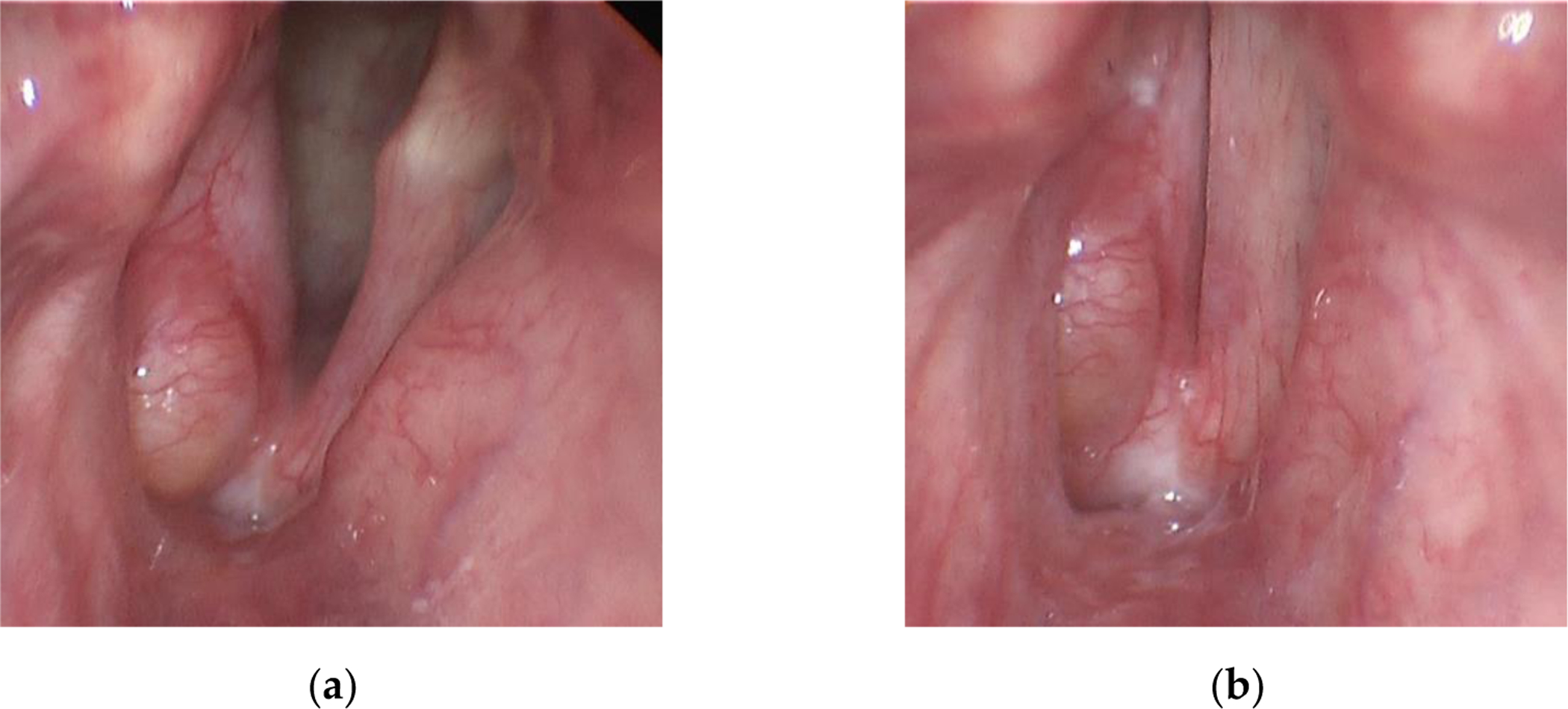
Endoscopic images of the larynx from the videostroboscopy examination of the participant who had previously undergone a unilateral (right vocal fold) hemilaryngectomy to treat laryngeal cancer. Shown are snapshots of the vocal folds in states of (**a**) abduction and (**b**) adduction.

A 78-year-old male was recruited for this study who had previously (12 years prior) undergone a unilateral, right vocal fold hemilaryngectomy for the treatment of laryngeal cancer (T2bN0M0 squamous cell carcinoma). An adipose tissue implant, taken from the periumbilical region, was injected to medialize the right vocal fold and facilitate glottal closure as the left vocal fold oscillated during phonation. The individual follows up regularly with his treating laryngologist for cancer surveillance and to assess vocal function, which includes laryngeal videoendoscopic examinations with stroboscopy. Figure 1 displays endoscopic images of the individual’s larynx in an abducted and adducted state. Video S1 (Supplementary Material) displays a segment from the videostroboscopic examination as the individual produces voice at a comfortable and higher-than-comfortable pitch level. According to clinical notes, the individual exhibited normal pliability and mucosal wave of the left vocal fold and diminished pliability and mucosal wave of the right vocal fold, consistent with expectations following the hemilaryngectomy and reconstruction. Complete glottal closure was observed during phonation with entrained vocal fold oscillation, mild phase asymmetry, and significantly reduced amplitude of the right vocal fold. There was no evidence of recurrent laryngeal disease.

### 2.2 Data collection

Following the clinical videostroboscopic assessment, the participant underwent a laryngeal high-speed videoendoscopic assessment with simultaneously recorded vocal function sensors. The endoscopist (a laryngologist who regularly performs outpatient transoral vocal fold injections) positioned the ISP probe with the left hand against the scar band on the right side of the larynx and synchronously recorded laryngeal high-speed videoendoscopy via a 70-degree transoral rigid endoscope (10 mm outer diameter; JEDMED, St. Louis, MO) held in the right hand. The high-speed camera was attached to the eyepiece of the endoscope (FASTCAM Model MC2, Photron, Tokyo, Japan) using a 45 mm–focal length lens adapter (PENTAX Medical) and high-speed videoendoscopy data were recorded with a Color High-Speed Video System (Model 9710, PENTAX Medical, Montvale, NJ). The high-speed video data were recorded at 2000 frames per second with maximum frame integration time (~0.5 ms) and a spatial resolution of 512 pixels ×512 pixels. The imaging light source consisted of a 300-watt short-arc xenon lamp.

Five channels of sensor data recorded by an acoustic microphone, electroglottograph, neck-surface accelerometer, and the two pressure sensors of the ISP probe were time-synchronized with the high-speed video data. The acoustic signal was recorded 15 cm from the lips using a head-mounted, omnidirectional microphone (model ME102, Sennheiser Electronic GmbH, Wedemark-Wennebostel, Germany). Electroglottography (Model EG2-PC; Glottal Enterprises, Syracuse, NY) provided a signal associated with vocal fold tissue contact that is high-pass filtered to reduce noise and low-frequency conductance changes. Neck-surface vibration was measured using an accelerometer (BU-27135; Knowles, Itasca, IL) that can be analyzed to yield glottal airflow estimates [37, 38] and has shown potential for long-term monitoring of vocal function [e.g., 39, 40–42]. The accelerometer was enclosed in a silicone epoxy and mounted onto the participant’s neck halfway between the sternal notch and thyroid prominence using Double Stick Discs (Model 2181, 3M, Maplewood, MN).

**Figure 2.**
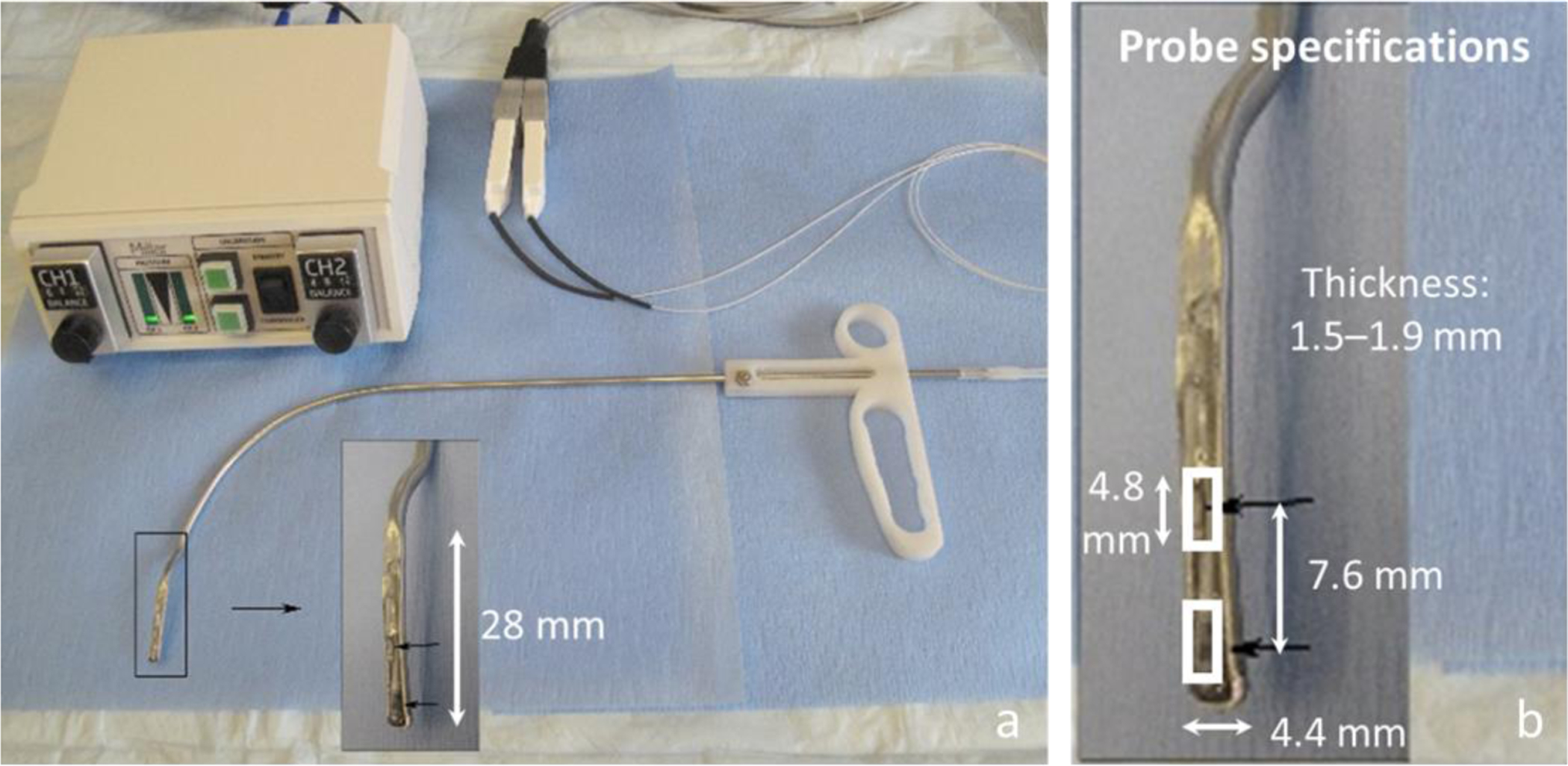
*In vivo* intraglottal/subglottal pressure (ISP) probe with two pressure sensors at the probe tip to simultaneously measure intraglottal and subglottal pressure during phonation, **(a)** ISP probe with a Ford injector–like handle and two-channel signal conditioning electronics, **(b)** zoomed-in view of the ISP probe tip showing dimensions of the two in-line pressure sensors. From [13].

Figure 2 displays a photograph of the dual-sensor ISP probe and its dimensions [13]. The width of the probe tip was 4.4 mm, and the probe tip length (before curvature began) was 28 mm. The thickness of the probe at the sensor locations was maximally 1.9 mm, which is larger than in previous devices [0.4 mm [9, 10] and 0.29 mm [8]] due to the dimensions of the pressure-sensing elements. The effect of the thickness of the probe tip was mitigated by the placement of the probe against the surgically reconstructed vocal fold (scar band) of the study participant as the contralateral vocal fold came into contact with the pressure probe. The two probe catheters were connected, via extender adapter cables, to a two-channel signal conditioning unit (PCU-2000, Millar, Inc.) that provides electrical isolation, analog knobs for zero control, and a flat frequency response of 0–1000 Hz. Measurement of intraglottal and subglottal pressures was accomplished using the two miniature, pressure-sensitive sensors of the ISP probe, respectively (Mikro-Cath Pressure Catheter, Millar, Inc., Houston, TX). The pressure sensing elements consisted of diffused, piezoresistive semiconductors with a dynamic range of −6.7 kPa to 40 kPa (−70 cm H_2_O to 400 cm H_2_O) and a flat frequency response up to 10 kHz. Each pressure transducer consists of an ovoid capsule that is 4.8 mm long and 1.17 mm in diameter, connected to a 120 cm long flexible cable. The sensing element measures 1.0 mm × 1.0 mm (sensing area of 1.0 mm^2^) and is recessed 0.37 mm into the cylindrical surface of the transducer; see Figure 2 in Motie-Shirazi, et al. [12]. To reduce the uncertainty in collision pressure measurements by the ISP probe, the sensors were embedded in medical-grade room-temperature-vulcanizing (RTV) silicone and covered with a thin 0.125 mm silicone sheet to create a flat contact surface [12, 13].

The five data channels were each digitized at 8000 samples per second and 16-bit quantization by a digital acquisition board (6259 M series, National Instruments, Austin, TX). Gain control and anti-aliasing filtering was set by preconditioning electronics (CyberAmp model 380, Danaher, Corp., Washington, DC). Time synchronization of the high-speed video data and data channels was accomplished by a common clock source from the National Instruments board that supplied the 2000 Hz (video) and 8000 Hz (data) sampling signals.

Two of the alternating-current (AC) signals were calibrated using linear scaling factors to convert from voltage levels to units of pascals for the microphone signal [43] and units of cm/s^2^ for the neck-surface accelerometer signal. The AC electroglottography signal was left uncalibrated as a relative measure vocal fold contact. Calibration of the ISP probe’s pressure sensors accounted for the sensor silicone enclosures by submerging the probe in a graduated cylinder filled with water and noting the voltage level at given submergence depths for each sensor (i.e., hydrostatic pressure) and computing a best-fit line to the data (coefficient of determination for the line was 1.0). The linear, multiplicative scale factors that mapped pressure sensor voltage levels (V) to units of pressure (cm H_2_O) were stable and repeatable at 1.596 (cm H_2_O)/V for the intraglottal pressure sensor and 1.722 (cm H_2_O)/V for the subglottal pressure sensor. The DC level of the intraglottal and pressure signals exhibited a low-frequency component (DC drift) also observed in prior *in vivo* studies that was associated with thermal fluctuations [8, 9]. Zero subglottal pressure was defined during the silence period prior to the onset of phonation (3.4 cm H_2_O correction factor was subtracted from the measured signal), and the intraglottal zero pressure level was defined at the most-negative peak pressure value [13] when the intraglottal sensor was positioned in the plane of vocal fold collision during phonation (1.3 cm H_2_O correction factor was added to the measured signal).

**Figure 3.**
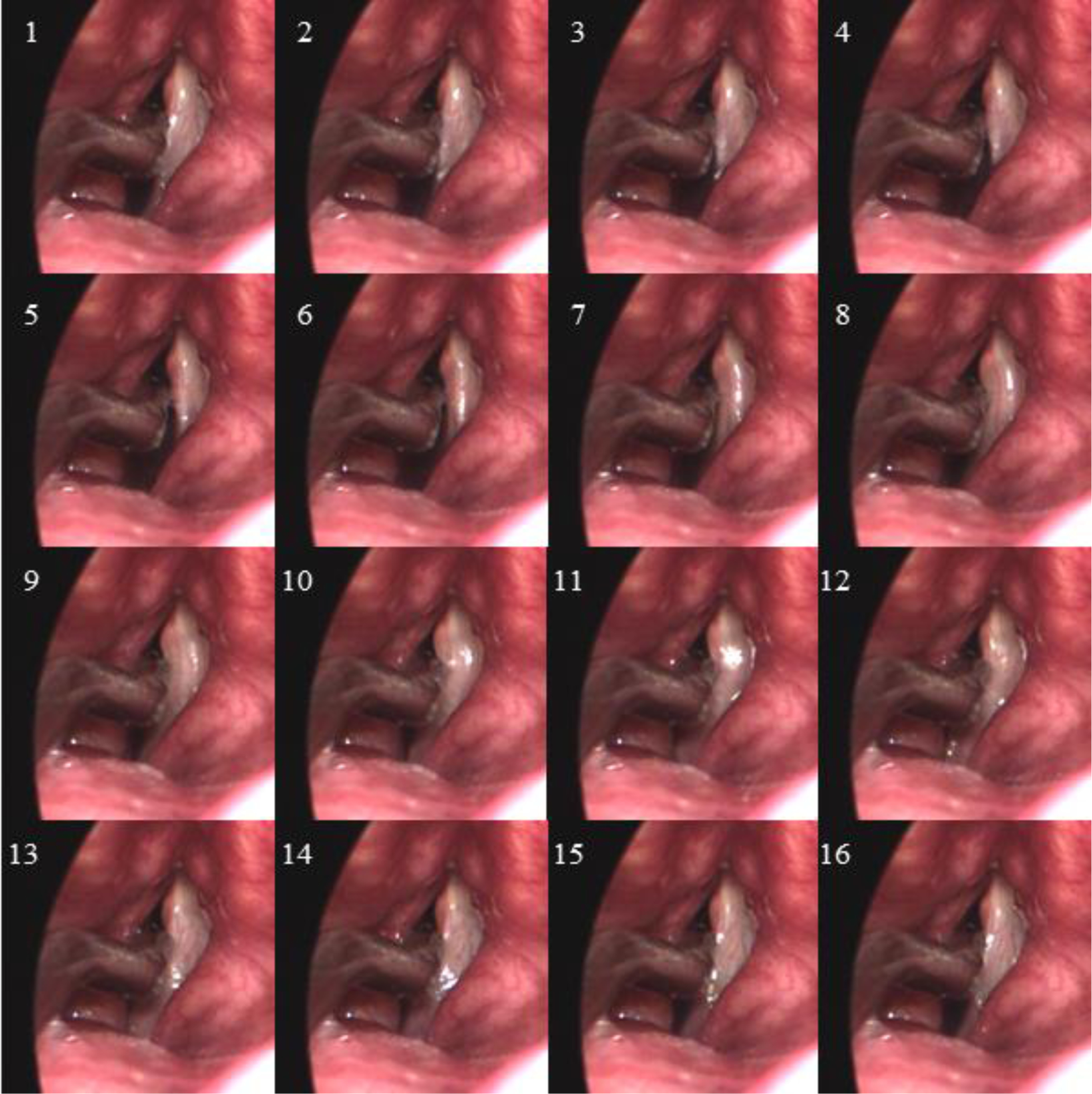
One phonatory cycle is displayed from the high-speed videoendoscopy data (2000 frames per second) during which the intraglottal pressure sensor of the ISP probe was deemed to be in the strike zone of the left vocal fold. Sixteen frames (frame indices indicated) are shown for the phonatory cycle that was 8 ms in duration, translating to a fundamental frequency of tissue oscillation of 125 Hz.

Endoscopic visualization of the larynx during sustained phonation at comfortable pitch and loudness levels allowed the endoscopist to place the dual-sensor pressure probe such that the proximal sensor was positioned intraglottally and the distal sensor subglottally. The clinician held a left-handed ISP probe with pressure sensing elements facing the functioning left vocal fold of the participant. As recommended in prior work [13], it was necessary to ask the subject to produce sustained phonation while the ISP probe was slowly swept in the superior-inferior dimension. Then, during the data analysis phase, features related to vocal fold collision were then identified to determine when adequate vocal fold contact occurred to capture peak collision pressures. Figure 3 displays high-speed videoendoscopic images from one phonatory cycle during which the proximal pressure sensor of the ISP probe was deemed to be positioned in the strike zone of vocal fold collision. Video S2 (Supplementary Material) shows the corresponding high-speed videoendoscopy data for this phonatory segment.

### 2.2 Data analysis

The proximal pressure sensor of the ISP probe was considered to be in the strike zone of vocal fold collision when the intraglottal pressure signal exhibited the three expected characteristics observed in the literature [e.g., 13]: an impulsive peak at the start of the closed phase, rounded peak during the open phase, and minimum value around zero immediately preceding the impulsive peak of the subsequent phonatory cycle. For phonatory segments exhibiting vocal fold collision, the peak collision pressure was defined as the maximum pressure value for each cycle. The mean subglottal pressure was computed from the simultaneously recorded signal of the distal pressure sensor of the ISP probe. The fundamental frequency of the intraglottal pressure waveform was computed using an autocorrelation-based method designed to process acoustic voice signals [44]. Vocal sound pressure level was computed from the root-mean-square of the calibrated acoustic microphone signal in dB SPL at 15 cm from the lips.

Two numerical modeling approaches were applied to validate their estimates of vocal fold collision pressure given the glottal area waveform and/or vocal fold kinematics derived from the high-speed videoendoscopy data. The first numerical approach employed a Hertzian impact model to estimate vocal fold collision pressures using vocal fold edge and glottal area contour analysis, referred to as contact pressure analysis [see 45 for algorithmic details]. This approach took vocal fold kinematic information extracted from laryngeal high-speed videoendoscopic data as input only. A Kalman filter scheme linked the vocal fold edge motion with a lumped-mass representation of vocal fold tissue contact mechanics. The lumped-mass model was used to estimate the non-physical overlap in the Hertz contact model associated with the deformation of two colliding cylinders. For both consistency and simplicity, the vibrating tissue of each vocal fold edge was represented by a parabola, with anterior and posterior anchor points that were selected for each vocal fold. Given that the tip of the ISP probe rested on the right vocal fold of the participant in this study (blocking the visualization of the vocal fold; see Figure 3), manual adjustment of the anchor points was performed such that the vibrating tissue of the left vocal fold collided at the probe location. Even though this contact pressure analysis method was originally designed to produce normalized contact pressure values, results in physical units were obtained by applying generic material properties for the vocal fold tissue; an effective Young’s modulus of 24.75 kPa and a Poisson ratio of 0.5 was taken from Table 2 in Díaz-Cádiz, et al. [45]. Note that this approach only provides point estimates of the peak vocal fold collision pressure. Alternative models are necessary to yield confidence intervals on these point estimates, as well as estimates of other important vocal function measures.

The second mathematical model accomplished the goal of deriving vocal fold collision pressure and other hard-to-measure physiological measures, such as subglottal pressure, glottal airflow, and intrinsic muscle activation levels. This model utilized a Bayesian estimation technique to analyze the spatially calibrated glottal area waveform to derive time-varying signals and confidence intervals for vocal fold collision pressure and these other physiological measures [see 46 for algorithmic details]. This approach was based on a subject-specific, body-cover model of the vocal folds [47] and an extended Kalman filter with the glottal area waveform as the only observation signal, i.e., Case I in Alzamendi, et al. [46]. The glottal area was obtained using the Glottal Image Explorer software [48], which also allowed for estimating the portion of the glottal area that was visually blocked by the ISP probe by adjusting the software parameters. To mimic the hemilaryngectomy condition of the left vocal fold coming in contact with the ISP probe, only the left vocal fold was analyzed, and a symmetric condition was assumed. Spatial calibration of the high-speed video was accomplished using the known dimensions of the probe tip as an imaging ruler. Model outputs were subglottal pressure, posterior glottal gap area, intrinsic muscle activation levels of the cricothyroid and thyroarytenoid, and vocal fold collision pressure. Each of the outputs was computed with 95% confidence intervals. Initial model conditions were set to yield a large initial uncertainty at the beginning of the signal to assess the speed of model convergence. For both models, the error was computed between the model-estimated peak vocal fold collision pressure and measured collision pressure from the ISP probe data.

## 3. Results

The intraglottal pressure sensor was deemed to be positioned as desired in the phonatory strike zone by comparing the intraglottal pressure sensor waveform with that of the subglottal pressure sensor. If the two sensor signals were positively correlated and in phase with each other, then both sensors were determined to both be positioned either subglottally or supraglottally. If the two sensor signals had significant waveform differences between them (with cycles occurring at the same fundamental frequency), then the two sensors were considered to be (desirably) in different locations within and around the glottis. The waveform of the (proximal) intraglottal pressure sensor was further investigated to determine if the expected waveshape characteristics, per the literature, were observed.

**Figure 4.**
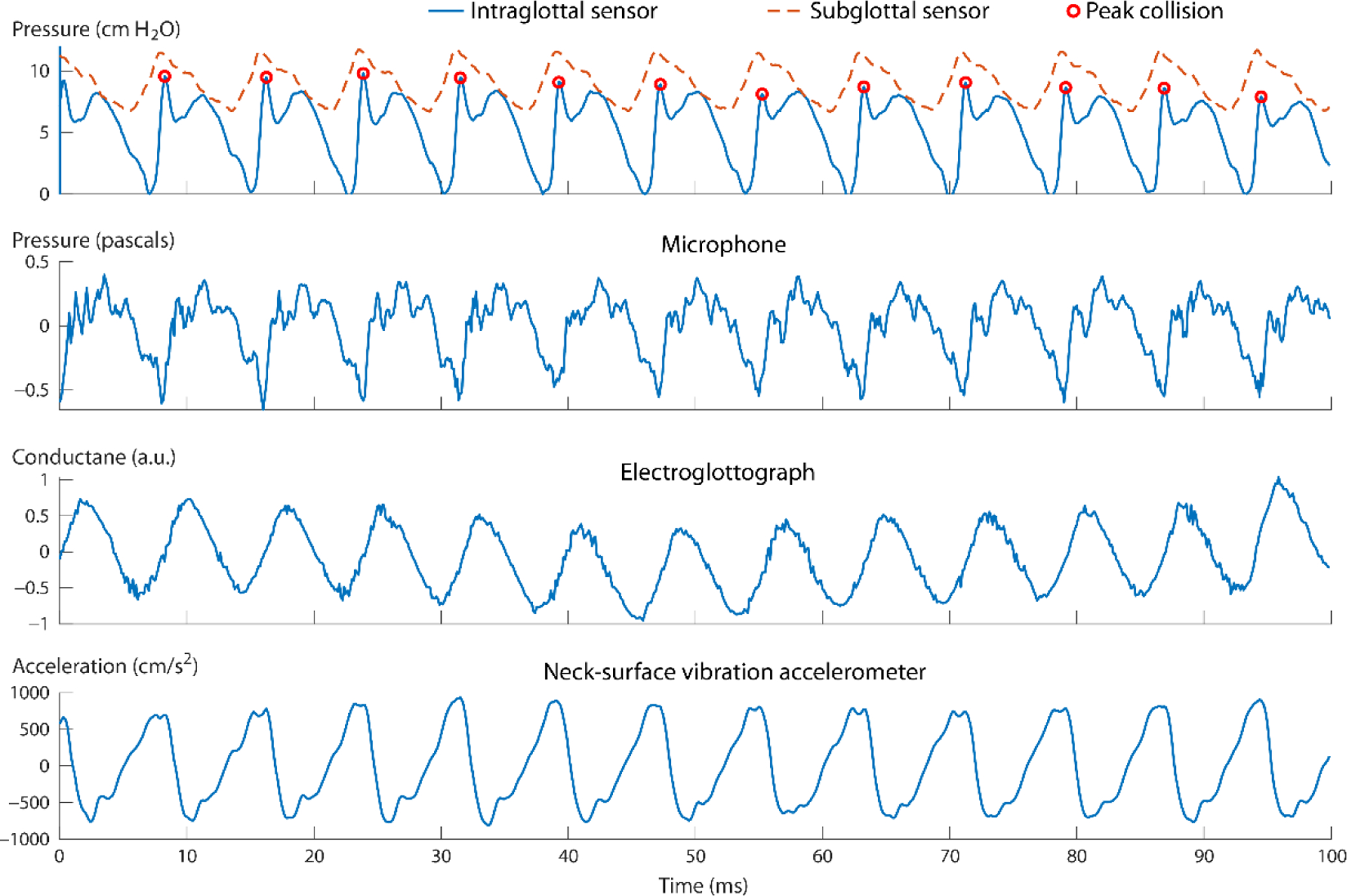
Calibrated signals recorded during a phonatory segment when the intraglottal pressure sensor was determined to be in the strike zone. Shown as circles are the peak collision pressures in the intraglottal sensor signal during each phonatory cycle.

Figure 4 displays the synchronized data signals during a 100-ms phonatory segment during which vocal fold collision was sensed by the intraglottal pressure signal. The vocal sound pressure level was 81.4 dB SPL re 15 cm, fundamental frequency was 126.1 Hz, mean subglottal pressure was 9.0 cm H_2_O, and the mean peak collision pressure was 9.0 cm H_2_O. The ratio between the peak collision pressure and subglottal pressure was thus 1.0, which is within the range of 0.5 to 3.8 that has been observed in excised hemilarynx experiments [13, 22]. We believe this is the first time that the expected intraglottal pressure waveform has been captured *in vivo* in a human speaker with the two expected waveform components due to impact pressure at the start of the closed phase (impulsive positive-going peak) and aerodynamic pressure during the open phase (more-rounded positive-going peak). The conductance signal of the electroglottograph corroborates the timing of the open and closed phases in relation to the intraglottal/collision pressure signal. The polarity of the accelerometer was such that motion away from the body (perpendicular to the neck surface) was recorded as positive-going amplitude values. The microphone signal captured the negative-going impulse per cycle that is associated with the excitation of the voice source by the maximum glottal flow declination rate [49]. Video S4 (Supplementary Material) shows an excerpt of this phonatory segment in a graphical user interface that displays the synchronized high-speed videoendoscopy with the multimodal sensor signals from the acoustic microphone, electroglottograph, neck-surface accelerometer, and the two ISP probe signals of intraglottal and subglottal pressure.

### 3.2. Validation of the two model-based estimates of vocal fold collision pressure

**Figure 5.**
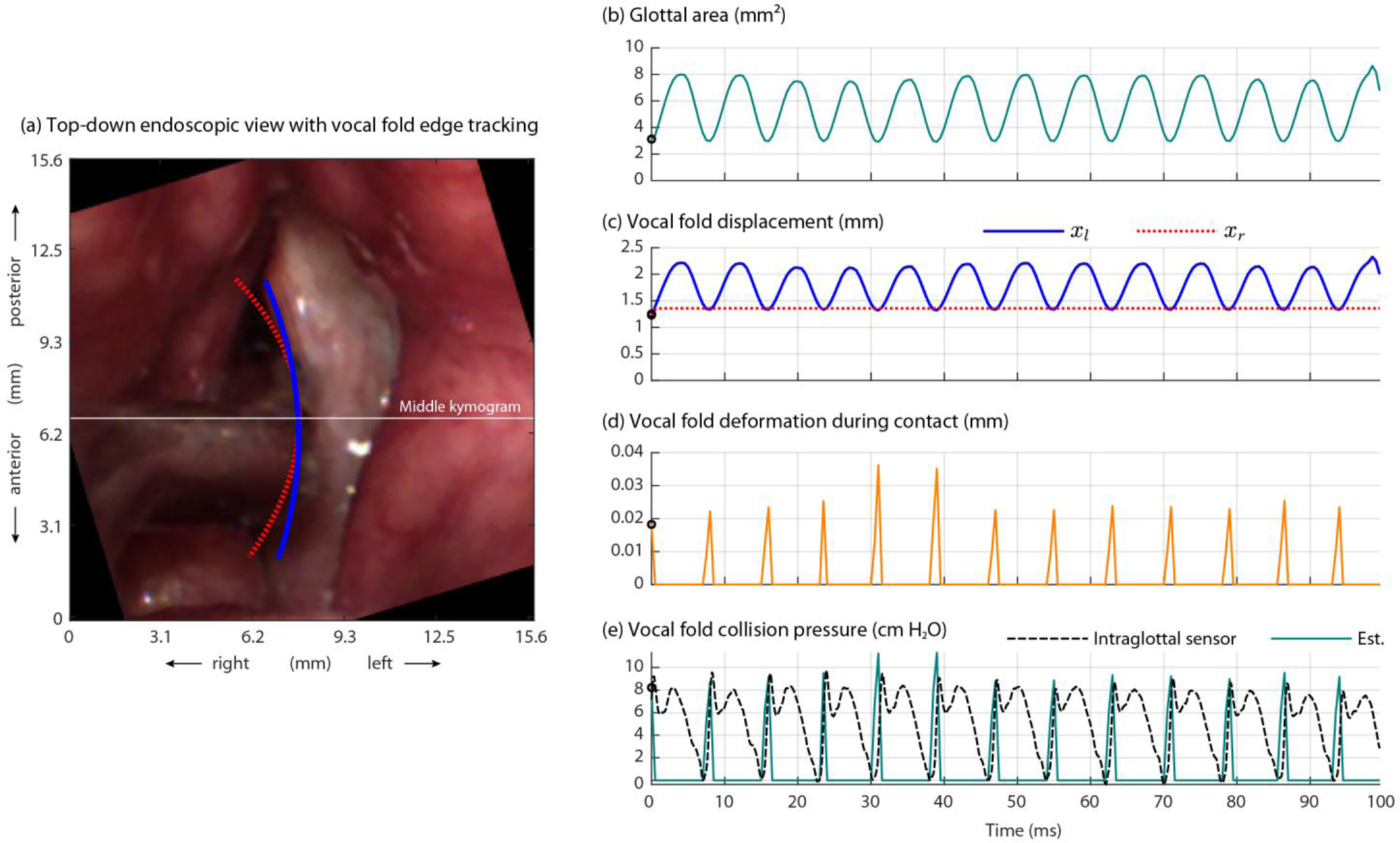
Results of the Hertzian contact pressure analysis method from Díaz-Cádiz, et al. [45]. Shown are the (**a**) first high-speed videoendoscopic frame with vocal fold edge tracking, (**b**) measured glottal area waveform, (**c**) vocal fold displacement at the middle digital kymogram of the left (*x*_l_) and right (*x*_l_) folds using a Kalman filter, (**d**) vocal fold deformation during contact (model output), and (**e**) vocal fold collision pressure as estimated by the model (Est.) and directly measured by the ISP probe’s intraglottal sensor.

Figure 5 shows the results of the contact pressure analysis method that incorporated a Hertzian model of collision [45]. Plotted are the input measured variables to the model from the high-speed videoendoscopy imaging data (calibrated glottal area and vocal fold displacement) and output estimated parameters from the model (vocal fold deformation at contact and vocal fold collision pressure). See Video S4 (Supplementary Material) showing a movie of Figure 5 that plots over time the input and output variables overlaid on the high-speed videoendoscopy frames. Direct comparison between the estimates of collision pressure from the model and the measured intraglottal pressure sensor signal is illustrated in Figure 5 as well. The impact stress component matches the first peak of the measured intraglottal signal well. The root-mean-square error between the estimated collision pressure peaks and the measured peak collision pressures across all cycles in this 100-ms phonatory segment was 1.04 cm H_2_O, corresponding to a mean absolute error of 0.81 cm H_2_O and mean absolute percentage error of 9.1%. The resulting contact pressure component is in good agreement with prior numerical studies, e.g., Fig. 4(a) in Tao, et al. [18].

**Figure 6.**
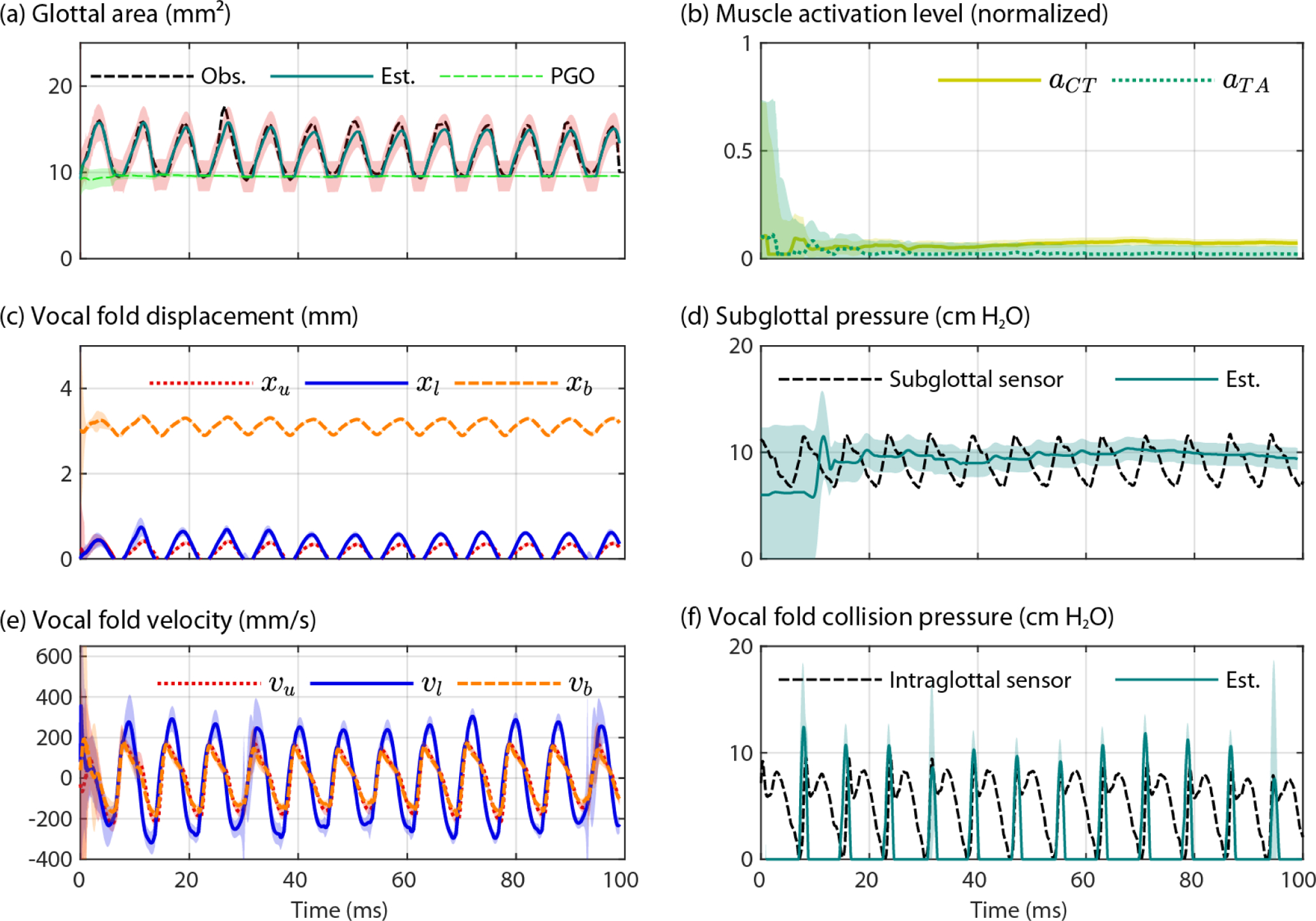
Bayesian estimation of physiological parameters of interest using the subject-specific modeling approach from Alzamendi, et al. [46]. Shown are the (**a**) observed (Obs.) and estimated (Est.) glottal area waveforms with estimated posterior glottal opening (PGO), (**b**) estimated muscle activation levels of the cricothyroid (*a*_*CT*_) and thyroarytenoid muscles (*a*_*TA*_), (**c**) measured vocal fold displacement for the upper (*x*_u_), lower (*x*_l_), and body (*x*_b_) masses of the left (vibrating) vocal fold, (**d**) subglottal pressure as estimated by the model (Est.) and directly measured by the ISP probe’s subglottal pressure sensor, (**e**) measured vocal fold velocity for upper (*x*_u_), lower (*x*_l_), and body (*x*_b_) masses of the left (vibrating) vocal fold, and (**f**) vocal fold collision pressure as estimated by the model (Est.) and directly measured by the ISP probe’s intraglottal pressure sensor. Shaded areas correspond to 95% confidence intervals around the model-estimated parameters.

Figure 6 shows the results of the second model that applied Bayesian estimation to a subject-specific, lumped-mass vocal fold model [46]. Plotted on the left panels of Figure 6 are the input measured variables to the model from the high-speed videoendoscopy imaging data: calibrated glottal area, vocal fold displacement, and vocal fold velocity of the medial edge. On the right panels of Figure 6 are the output estimated parameters from the model: normalized muscle activation levels of the cricothyroid and thyroarytenoid muscles (*a*_*CT*_ and *a*_*TA*_, respectively), subglottal pressure, and the vocal fold collision pressure signal. In addition to the parameter estimates, Bayesian estimation also provides the confidence intervals associated with each output parameter waveform. The large initial model uncertainty within the first 10 ms was expected due to the initial conditions applied; following that transient period, the confidence interval of the model outputs rapidly decreased and reached steady-state values, illustrating good model convergence with tight confidence intervals. As found in direct measurement, an approximate 1:1 relation between the model-based peak collision pressure and subglottal pressure values was observed using the Bayesian estimation method. The root-mean-square error between the estimated vocal fold collision pressure peaks and the measured peak collision pressures was 1.82 cm H_2_O, corresponding to a mean absolute error of 1.58 cm H_2_O and mean absolute percentage error of 17.9%.

## 4. Discussion

This study was the next step in ongoing work to demonstrate the feasibility of obtaining direct measurements of the vocal fold collision pressure during phonation in an individual that was specially selected to reduce the challenges of performing the procedure in a typical individual with bilaterally vibrating vocal folds. The synchronized collection of estimates of subglottal air pressure below the vocal folds was necessary to effectively interpret the impact of aerodynamic versus tissue collision forces on the pressure measurements being obtained at the vocal fold level. The simultaneous recording of high-speed videoendoscopy and non-invasive recordings of neck-surface accelerometry, acoustics, and electroglottography allow for the cross-correlation these measures with the intraglottal/subglottal pressure signals of the ISP probe. Such data are much more valid and valuable if they can be reliably acquired *in vivo* as opposed to the excised situation where there is no neural innervation of associated flaccid muscles and no perfusion (blood supply) to the muscles and phonatory mucosa. These data are also critical for improving our ability to use and validate computer models of vocal fold phonatory function that will provide better insight into the underlying biomechanics associated with normal and pathological voice production, and ultimately help guide the design of improved prevention, diagnostic, and treatment approaches.

### 4.1 Direct measurement of intraglottal/colliion pressure waveform characteristics

To the authors’ knowledge, the results of this study represented the first time that direct measurement of *in vivo* vocal fold intraglottal/collision pressures resulted in expected waveform characteristics in a human speaker. In previous work [8], it was found to be more challenging to place a sensor between two vibrating vocal folds in a vocally healthy individual than it was to perform this procedure in an individual with one functional vocal fold (following a hemilaryngectomy surgical procedure). This previous proof-of-concept study demonstrated that it was possible to safely acquire estimates of vocal fold collision forces in one vocally healthy individual (with two functional vocal folds) and three individuals with a hemilaryngectomy. Conclusions from that work were that: 1) simultaneous measurement of subglottal pressure is necessary to accurately interpret the vocal fold collision data, and 2) measures for the individuals with a hemilaryngectomy were easier to obtain and were more reliable and valid than those obtained for the vocally healthy speaker. However, the intraglottal force sensor used in that study had a relatively large sensing area and yielded triangular waveforms that did not appear to exhibit the signature impulse-like component at the time instants of vocal fold collision (an electroglottography signal provided verification the instants of glottal closure).

Building on this previous work, in the current study, verification of the correct placement of the intraglottal pressure sensor was accomplished using novel, concurrent recordings of laryngeal high-speed videoendoscopy and a subglottal pressure signal. Our prior investigations of a hemilarynx model took advantage of a dual high-speed videoendoscopy setup that offered visual verification of sensor placement from a top-down, endoscopic view and a medial, *en face* visualization of the of the hemilarynx setup [13]. That work concluded with the following guidelines for using the ISP probe *in vivo*:

1. Endoscopic visualization is necessary to guide placement of the ISP probe such that the distal pressure sensor at the probe tip is positioned subglottally and the proximal sensor is positioned in the glottis in the phonatory strike zone to sense vocal fold impact collision pressure during phonation.
2. In individuals with a hemilaryngectomy, the ISP probe should rest on the medialized scar band that replaces the excised vocal fold tissue, such that the pressure-sensing element comes into direct contact with the functioning vocal fold.
3. The positioning of the intraglottal pressure sensor is in the phonatory strike zone if the following waveform characteristics are exhibited:
  - An impulsive peak in the direction of increasing pressure at the instant of vocal fold contact;
  - A more-rounded peak following the impulsive peak that senses aerodynamic pressure build-up during the open phase; and
  - A minimum value approaching zero or negative pressure immediately preceding the impulsive peak of the subsequent phonatory cycle, reflecting rapidly decreasing intraglottal pressure as airflow accelerates.

**Figure 7.**
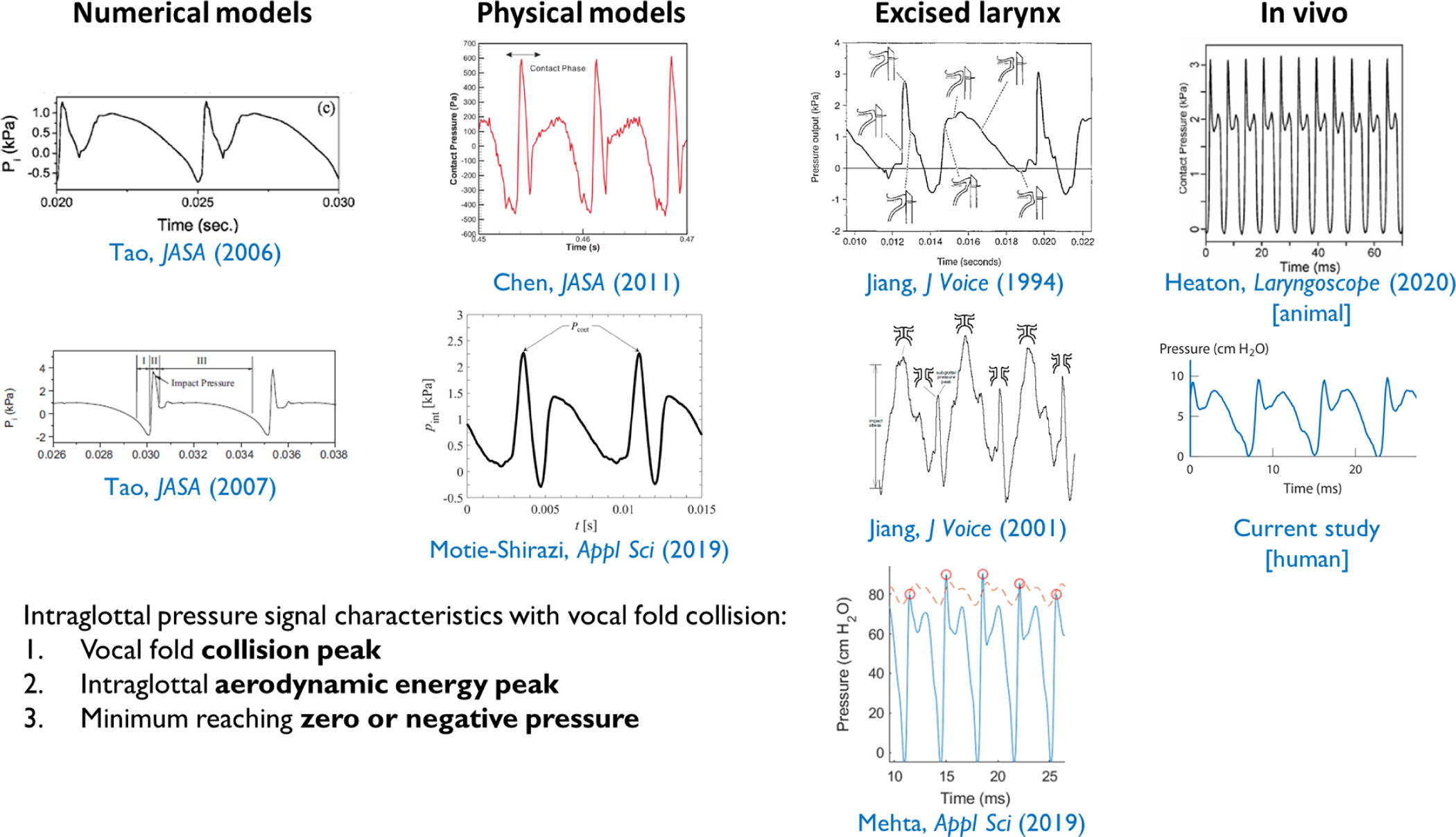
Comparison of the intraglottal pressure signal during phonation of the current study with exemplary intraglottal pressure signals observed in the literature according to numerical [Figure 4c, 18, Figure 3a, 19], physical (synthetic material) [Figure 14, 12, Figure 7 [right], 14], excised larynx [Figure 10i, 13, Figure 7, 22, Figure 5, 25], and *in vivo* animal [Figure 6A, 29] studies. Figures reused with permission.

Figure 7 displays exemplary intraglottal pressure waveforms observed in the literature according to numerical, physical, excised, and *in vivo* animal models of phonation. The expected waveform characteristics were observed during the recording in this study as shown in Figure 4.

### 4.2 Validation measures for numerical models of voice production

Although using the ISP probe is not practical for routine data collection, the data collected from the direct measurement of intraglottal, collision, and subglottal pressures using the ISP probe in a select group of individuals can be used as references in numerical models that can be optimized to estimate these important parameters in patients with voice disorders or vocally healthy speakers. Two numerical modeling approaches were tested in this paper to validate model-based estimates of vocal fold collision pressure using only data from the laryngeal high-speed videoendoscopy recording, a procedure that is feasible to perform in practice. Good agreement was exhibited between the model-estimated collision pressures and measured vocal fold collision pressures. This agreement provides valuable first *in vivo* validation of the vocal fold collision pressure estimates derived by these types of models, which is critical, as these engineering methods are expected to be more easily translated into clinical practice.

To avoid confirmation bias and given the pilot nature of this study, further *in vivo* validation is needed to assess the robustness of the indirect estimation approaches. However, prior validation against silicone vocal fold models and prior studies have been successfully performed for both modeling approaches utilized in this study [45, 46]. It is interesting to note that both numerical and experimental findings point to a close relation between the peak contact pressure and the driving subglottal pressure, near a 1:1 ratio. The assessment of this relation is of clinical relevance and requires further attention, as other factors are hypothesized to influence this relation, such as muscle activation level, loudness condition, mode of vibration, and supraglottal compression.

### 4.3 Clinical implications

One of the most common causes of voice disorders is phonation-related trauma to vocal fold tissue which can cause the loss (e.g., scarring) of normal superficial lamina propria and/or the formation of benign lesions on the vocal folds (e.g., vocal fold nodules). Such damage to the phonatory mucosa is believed to be associated with excessive perilaryngeal muscle activity, termed vocal hyperfunction; however, the actual underlying mechanisms that produce varied vocal fold traumatic injury are poorly understood. The direct measurement of vocal fold collision pressures during phonation, in addition to frictional shearing stresses on vocal fold tissue and dissipated energy dose [50, 51], has important implications in understanding the role of vocal fold collision in the etiology and pathophysiology of phonotraumatic vocal hyperfunction [3]. It is believed that a primary contributing factor to phonotrauma is an increase in level and/or duration above safe thresholds of the collision forces that are generated by the vibrating vocal folds during voice production. However, valid measures of vocal fold collision forces are lacking/incomplete, particularly with respect to what constitutes safe versus damaging levels of such forces.

Of particular interest is the development of measures that can be applied to ambulatory voice monitoring technologies, with the ultimate goal to better differentiate what constitutes healthy versus damaging vocal function. Real-world voice monitoring devices often use sensors placed on the surface of the neck below the larynx as a way to track features of voice production in a way that is confidential, non-obtrusive, and robust to environmental noise artifacts [40, 41, 52, 53]. The vibration accelerometer used in the current study was similarly placed on the neck surface above the sternal notch and below the thyroid prominence to mimic the sensor positioning of an accelerometer used for ambulatory voice monitoring. Several recent studies have started to elucidate vocal features and behaviors during daily life that may be associated with the presence of hyperfunctional voice disorders [39, 54–58]. Traditional in-field accelerometer features include estimates of sound pressure level [59] and fundamental frequency that have been combined with voice activity detection to yield vocal dose measures that are designed to indirectly quantify the accumulated effects of phonotrauma [60]. Later formulations of vocal dose measures have attempted to incorporate the effects of vocal fold collision [50], which is important since phonotrauma is hypothesized to be caused by repetitive stress on the mid-membranous portion of the vocal folds [5–7]. Since the data in this study point toward a potential 1:1 correspondence between vocal fold collision pressure and subglottal pressure in certain scenarios, we can take advantage of work aimed at estimating subglottal pressure from neck-surface vibration and apply this analysis to ambulatory voice signals [61–64].

### 4.4 Study limitations

It is acknowledged that results should be tempered by the limited analysis of data from a single individual. Given the unique nature of the recording setup, the fact that vocal fold collision characteristics were captured in the intraglottal pressure signal was encouraging. Introducing a probe into the glottis has the potential to disrupt the natural oscillatory mechanisms of the vocal folds. Figure 3 showed the size of the tip of the ISP probe in relation to the size of the glottis and surrounding vocal fold tissue; anterior and posterior glottal gaps may have been introduced due to the presence of the ISP probe. However, despite the introduction of the ISP probe, the study participant was still able to sustain a vowel during the endoscopic procedure and produce relatively stable phonation. Future work is needed to study the effects of varying degrees of loudness levels, pitch conditions, and glottal configurations leading to breathy, modal, and pressed phonation.

Previous work with bench-top physical models of the vocal folds have suggested that caution should be used when interpreting waveform signatures of the intraglottal pressure sensor signal [12]. There could be some uncertainty with respect to whether the intraglottal sensor waveform in Figure 4 were actually from the vocal fold coming into contact with the sensor, or whether the sensor was slightly superior to the strike zone and actually sensing acoustic pressures. In addition, the pressure sensors exhibited signal drifts in the DC level that were also observed in previous studies [8, 9]. We believe this drift is primarily caused by thermal energy fluctuations in the glottis due to internal body temperature and the use of a bright xenon light source necessary for high-speed videoendoscopic imaging. Future work could address thermal energy changes by pre-heating the tip of the ISP probe in hot water to match the internal temperature of the glottis.

## 5. Conclusions

The goal of this study was to illustrate feasibility of the direct measurement of *in vivo* vocal fold collision pressures during phonation using the recently developed dual-channel ISP probe. The results successfully demonstrated this feasibility in an individual with a hemilaryngectomy, motivating ongoing data collection that is designed to aid in the development of vocal dose measures that incorporate vocal fold impact collision/stress. Even though further *in vivo* validation with more subjects is needed, the good agreement in this case study between the vocal fold modeling methods and the experimental results illustrates the potential of the modeling tools for both providing access to additional measures of vocal function that are difficult to obtain and advancing the understanding of the underlying control mechanisms of normal and pathological voice production.

## Supplementary Material

Supplementary Material **Supplementary Materials:** The following are available online at www.mdpi.com/xxx, **Video S1:** Laryngeal videostroboscopy of the study participant with a right hemilaryngectomy during sustained phonation at a comfortable pitch and higher pitch. **Video S2:** Laryngeal high-speed videoendoscopy of vocal fold vibration (2000 frames per second) for the 100-ms phonatory segment during which the intraglottal pressure sensor was in the strike zone. **Video S3:** Graphical user interface displaying the high-speed video of vocal fold vibration (2000 frames per second) and time-aligned signals (8000 Hz sample rate) recorded by an acoustic microphone, electroglottograph, neck-surface vibration accelerometer, intraglottal pressure, and subglottal pressure. **Video S4:** Vocal fold edge contour displayed on the high-speed videoendoscopy data for the Hertzian contact pressure model, along with the input and output variables of the model. **Signals S5–S9:** The time-aligned signal data in WAV format for visualizing the signal waveforms during the 100-ms phonatory segment when vocal fold collision was detected by the intraglottal pressure signal: Neck-surface vibration acceleration signal (ACC; **S5**), electroglottography signal (EGG; **S6**), ISP probe intraglottal pressure sensor signal (IGP; **S7**) capturing vocal fold collision pressures during the phonatory closed phase and aerodynamic intraglottal pressure during the phonatory open phase, acoustic microphone signal (MIC; **S8**), and subglottal pressure signal (SGP; **S9**).

## Data Availability

The data presented in this study are available in the Supplementary Material section.
